# Pull me – push you? The disparate financing mechanisms of drug research in global health

**DOI:** 10.1186/s12992-024-01019-x

**Published:** 2024-02-19

**Authors:** Max Alexander Matthey, Aidan Hollis

**Affiliations:** 1 Department of Philosophy, Politics and Economics, Witten / Herdecke University, Alfred-Herrhausen-Straße 50, 58455 Witten, Germany; 2 Department of Economics, University of Calgary, 2500 University Drive NW Calgary Alberta, T2N 1N4 Canada AB Calgary,

**Keywords:** Pharmaceutical research, Neglected diseases, Policy making, Pull mechanisms

## Abstract

**Background:**

There is an inconsistency in the way pharmaceutical research is financed. While pull mechanisms are predominantly used to incentivize later-stage pharmaceutical research for products with demand in the Global North, so-called neglected diseases are chiefly financed by push funding. This discrepancy has so far been ignored in the academic debate, and any compelling explanation for why we draw the line between push and pull at poor people is lacking.

**Main body:**

Clinical development of new pharmaceuticals is chiefly financed by free market pull mechanisms. Even in cases where markets fail to deliver adequate incentives, demand enhancement mechanisms are used to replicate pull funding artificially, for example, with subscription models for antibiotics. Push funding in clinical research is almost always used when the poverty of patients means that markets fail to create sufficient demand. The general question of whether push or pull generally is the more efficient way to conduct pharmaceutical research arises.

**Conclusions:**

If the state is efficient in directing limited budgets for pharmaceutical research, push funding should be expanded to global diseases. If private industry is the more efficient actor, there would be enormous value in experimenting more aggressively with different approaches to enhance market demand artificially for neglected diseases.

## Introduction

Is it better to “pull” pharmaceutical innovation towards a goal through market-based rewards or to “push” it towards priority indications through subsidies? The push approach has been used intensively to address neglected diseases, while pull mechanisms dominate elsewhere—especially for clinical trials. This question is most relevant for the $4bn of annual research spending on neglected diseases [1]. For these diseases, the lack of effective demand results in a failure of regular commercial incentives to operate effectively. Investment into treatments for these diseases depends on public or charitable funding, and, unlike with global diseases, research on neglected diseases is almost entirely funded on a “push” basis. Where existing markets are inadequate, it is also possible to use demand enhancement mechanisms that effectively increase the incentives for industry to invest in innovation, creating a “pull” incentive.

The puzzle we address in this paper is the split in approaches. If “push” is genuinely more efficient, why is it not widely used to bring products to the point of market approval for global diseases? This is the argument advanced, for example, by the World Health Organization’s Council on the Economics of Health for All, which claims that such an approach would avoid “excessive financialization” and could direct research to the areas of greatest need, while ensuring access to all at affordable prices [2].

On the other hand, if “pull” is more efficient, it should be used wherever possible. As we discuss below, pulling innovation forward by allowing firms to exploit whatever market power their patents offer is the preferred choice by rich countries for the diseases that mainly affect affluent populations, and there is relatively little push funding for clinical development to bring drugs to market. Even in cases with inadequate effective market demand, high-income countries' approach appears to use demand enhancement rather than push funding to bring new medicines to market. Demand enhancement mechanisms are a way of creating artificial demand, enabling the use of pull funding to motivate for-profit drug development.

We begin by reviewing the roles of public and private funding in pushing and pulling drug development and manufacturing and then examine the theoretical merits of each. While theory doesn’t offer an answer to the question of what is preferred, we do observe that high-income countries choose to use pull for themselves but push for others.

## Background

Bringing a drug to market consists of numerous steps. Research prediscovery, typically performed in academic or other research institutions, is typically funded through government or foundation grants since the underlying key research cannot easily be patented [3]. Discovery preclinical includes target validation, compound screening, and lead optimization and is more likely to be funded privately, often in university spin-offs, though often there is a contribution from public funding [4]. Usually, this process takes several years. In vitro / in vivo testing is also typically privately funded. Clinical trials, lasting several years, are primarily privately funded. Regulatory submissions, manufacturing, and post-approval trials are almost always privately funded.

**Fig. 1:**
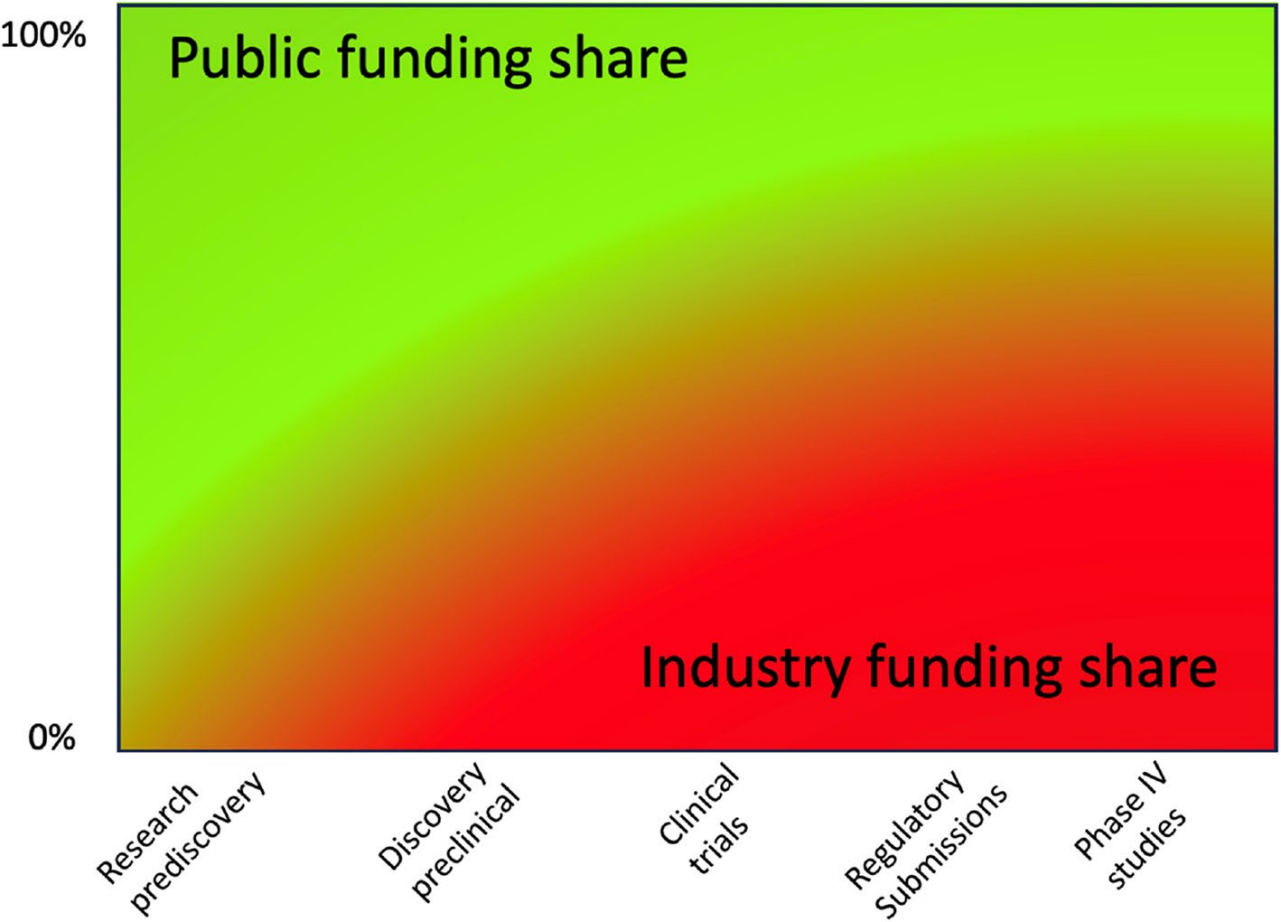
Evolution of Funding Shares

According to the Organisation for Economic Co-Operation and Development (OECD), industry investment in pharmaceutical research and development is approximately twice as large as the public contribution [5]. However, this ratio naturally depends on what research spending is included. The figure illustrates how the share of industry investment in bringing a product to market tends to increase as the product gets closer to approval (or beyond it) (Fig. 1).

While public funding tends to be dominant in the earliest stages of drug development – indeed, most research prediscovery tends to occur in academic institutions –, its presence tends to be less common in later research, especially at the post-approval stages [6]. Vieira et al. (2023, Fig. 12) show that commercial control and funding of clinical trials increases markedly from Phase 1 to Phase 3, although there continues to be some non-commercial sponsorship [7]. It is likely that almost all *pivotal* trials, i.e., those that are designed to demonstrate effectiveness of the medicine for regulatory approval, are funded by commercial interests [8].

Financial support, however, can come in many guises. While direct public funding, in which the funder chooses the project to be funded, is used at the pre-discovery stage, there are other ways of providing financial support. Notably, financial support can be given through tax subsidies: for example, the US Orphan Drugs Act provides a 50% tax credit for expenditures on qualifying clinical trials [9]. This type of financial support differs from direct funding in that orphan drug tax credits are not *directed* by the US government; instead, any firm with a qualifying clinical trial is eligible for this support. The most common form of public funding in support of drug development, however, is public drug insurance. For example, even in the US, public drug insurance covers over 40% of prescribed drug expenditures [10]. Many public insurance schemes use thresholds, so that reimbursement depends on cost-effectiveness. Such insurance is a pay-for-performance mechanism in which the firm is rewarded only if its product is found to be of value. Thus, the question on which we are focused is not whether government should provide financial support to the development of drugs, or which entity should own the research labs, but whether the funding mechanisms should use a push or pull approach.

Some recent innovations in this area have provided novel incentives to help bring products to market. The Advanced Market Commitment used by Gavi – the Vaccine Alliance was designed to support the manufacturing and delivery of pneumococcal vaccines for low-income countries by providing contracted subsidies upon purchase. This is, in effect, a form of public funding that is focused on the last stages in the value chain, which is not unusual in vaccine procurement; the Advanced Market Commitment is different only in that it offers *committed* funding to support the roll-out of vaccines in countries which are otherwise unable to purchase at high prices. Its original idea, however, was to support innovation from product development to delivery [11]. Similarly, the Antibiotic Subscription Pilot in England and Wales commits public money for market entry rewards up to £10 m per year for new antibiotics, subject to the products bringing adequate value to the market. This program, however, requires that the product be provided at no additional cost to the National Health Service.

The use of public funding to support drug development thus occurs throughout the value chain but tends to differ in character: public funding prior to clinical trials tends to be “push” funding, in which the funder commits financing based on the reputation of the research team and the interest of the project; public funding after clinical trials tends to be “pull” funding, in which there is payment only to reward actual success.

This general pattern is not observed when it comes to treatments for neglected diseases, which tend to be funded on a push basis from beginning to end. Neglected Tropical Diseases, as defined by the World Health Organisation, are a relatively small group of infectious tropical diseases within a broader group of neglected diseases receiving relatively little attention [12]. Generally, the rule is that the lower the average income of patients, the less investment there is in clinical trials [13]. Thus, even high-profile diseases such as malaria and tuberculosis receive relatively little investment and are in some sense “neglected.” (Indeed, the $4bn annual expenditures on R&D on neglected diseases, including malaria and tuberculosis, remains a very small fraction of global pharmaceutical R&D [1]). Private sector pharmaceutical R&D spending in OECD countries totals approximately $128bn annually [14]. For these neglected diseases, push funding is used even for clinical trials. The natural reason for this is that the people who suffer from these diseases tend to be poor and unable to pay high prices. Their lack of ability to pay results in a broken pull mechanism in the market, and push tends to be used all the way.

As described above, some commentators have strong objections to the current model of the pharmaceutical market, which is dominated by pull. If they are correct, and the standard pull model is *less* efficient, investing more in public funding of clinical research across all diseases would be better. On the other hand, if the standard pull model is *more* efficient, it would be better to use pull funding for neglected disease research, just as for other diseases.

### Overview of incentives and information flows

In this section, we examine the differences in incentives and information between the pull and push approaches. Since Product Development Partnerships (PDPs) are the most common institutional form used for push funding of late-stage clinical development, we focus on them. PDPs are non-profit entities funded mainly by philanthropies and aid agencies to develop new drugs [15]. They typically focus on a specific therapeutic area: for example, the Global Alliance for TB Drug Development and Medicines for Malaria Venture target clinical developments only within their focal areas [16]. PDPs typically work with commercial drug companies, sometimes splitting the market. (See, for example, the complex licensing framework between the TB Alliance, Janssen, and Mylan [17]). For example, the rights may be separated geographically, with the commercial partner retaining the rights to exploit the product in high-income markets only. The development of the product will typically be supported through push funding, with commercial partners having little or no expectation of significant revenues. In general, the PDP will not engage in the manufacture or sale of the product but will transfer the required rights to manufacturers with commercial limitations on pricing [18]. While funding for PDPs has been on a declining trend in real terms over the last 15 years, dropping from over $700 m to $433 m [1], there continue to be ambitious proposals for public financing of drug development. For example, Zimmerman et al. (2023) propose a $43 billion “aggregator” to organize and fund Phase 3 clinical trials for human immunodeficiency viruses, malaria, tuberculosis, and pneumonia over 12 years [19].

In the commercial model, public funding is, at best, partial and typically does not constrain pricing or exploitation of monopoly rights. Generally, most of the required financing is raised commercially, and firms take on the risk of investing in the product with no certainty of success. Often research through Phase 1 clinical trials is undertaken by small biotech companies whose end goal is to be purchased by one of the “big pharma” companies that have the capital to bring the product to market. If a product achieves regulatory approval, the firm will price it to maximize its profits, subject to restrictions on pricing imposed by payers. The critical difference, of course, is that in the commercial model, the funding is provided by investors hoping for profits from future sales.

The incentives and information flows are quite different. The PDP typically has excellent information about most of the projects that are sufficiently progressed for having a reasonable chance of advancing to the market within its portfolio. The reason is that the PDP offers to fund products that have no alternative prospects for commercial success. In contrast, in the commercial model, firms may have incomplete information about their rivals’ projects, at least until those projects enter clinical trials. Even then, much information – whether positive or negative – will be confidential. The wider information set about the alternative potential drugs in discovery possessed by PDPs is a clear advantage over the commercial model. As clinical trials proceed into Phases 2 and 3, however, it seems likely that some of this advantage is erased since securities laws mandate the disclosure of information of material importance to shareholders, which would include at least top-line information about trial outcomes.

It is unclear whether the same information advantage holds within the PDP. The challenge is that a PDP typically must decide between competing proposals from different product-development teams, each with different interests. Each team will want to emphasize the attractions of its own proposal. To be sure, the same process plays out within a firm, with committees deciding which projects should receive scarce capital. Unlike those in PDPs, these committees make decisions across multiple therapeutic categories, which gives them a significant efficiency advantage. If all the projects available to decision-makers are in one therapeutic area, their choice is intrinsically limited by the opportunities in that area. For example, a heavy investment in Alzheimer's Disease failed to bring effective new drugs to market for many years, with the limiting factors being a poor understanding of the underlying disease process. Any firm specializing in developing drugs for this challenging indication was likely to choose only among unprofitable opportunities. However, with a portfolio of products spread across different diseases, a firm can select from a range of alternative investment opportunities.

The commercial model also takes advantage of disaggregated information. PDPs can help to bring all players to one table, creating the opportunity to fund the best projects. In contrast, the commercial model is disorganized, which is a disadvantage in that poor projects may be funded by firms that do not know about superior alternative projects within the disease space. On the other hand, having a single organization choosing projects within a disease space may be burdened by “groupthink” [20]. If the decision-making leadership of the PDP decides which projects it will fund, the scope of projects will necessarily be limited. In contrast, multiple commercial organizations acting independently will likely have competing and heterogeneous decision processes that will support the funding of heterogeneous projects.

A further distinction between the competitive commercial model and PDPs is that competing firms may end up “racing” to get to market first. In many cases, a fundamental breakthrough enables a set of different drugs that are developed independently but around the same time [21]. Firms will then race to get their products through to approval since the first to market may benefit from early entry. In some cases, there may also be a race to patent. The effects of racing are ambiguous. On the one hand, racing benefits patients by making new drugs available to them earlier. On the other hand, racing may be wasteful if firms duplicate expenditures or invest excessively in trying to accelerate product development [22, 23]. In pharmaceutical markets, this may result in clinical trials that are shorter than optimal, potentially masking safety issues related to long-term use of some medications. This may also distort the set of diseases for which firms develop new drugs. For example, there is evidence that firms have prioritized investment in drugs for late-stage cancers, which can be trialled with a relatively short duration, over drugs for early-stage cancers, requiring longer trials to assess the survival effect [24].

The incentives within these organizations differ markedly. Success in the commercial model can yield enormous gains for the individuals involved, especially compared to the opportunities available within the PDP model. This has mixed effects, to be sure, with the incentives to achieve success creating support for investment but sometimes driving poor or even immoral behaviour in some situations [25]. The pharmaceutical industry has become notorious for “financialization”: many firms appear to have an emphasis on maximizing returns to shareholders, which allows them to pay extraordinary executive compensation and to fund large share buybacks [26].

One further difference between push and pull funding is the control that the funders exercise over who receives financial support. With pull funding, success in treating the disease is the main requirement for getting paid. This means that with pull funding, the funder lacks the ability to control who earns revenues. In contrast, with push funding, the funder can choose the exact recipient of funding. Thus, for example, a donor government seeking to support research on some neglected disease may choose to target its grants to domestic researchers, even if they are not the most productive globally. Not surprisingly, from a political perspective, the interest in providing domestic benefits to researchers and universities in high-income countries often dominates the interests of sick patients in poor countries. (Perhaps this helps to explain the low level of financial support for PDPs from national governments: PDPs typically do not direct their research spending for political gain). Some commentators have criticized PDPs as too reliant on “the dominant neocolonial structure of funding and operation” ([27], p.1017), and as ensuring that the “financial benefits … go almost exclusively to business and universities in the countries that provide the funding" ([28], p.57).

It is not clear from either the informational differences or the incentive structures whether push or pull funding is more effective in delivering results. However, we note that different approaches have been used in different situations. For products with large global markets, the commercial model in which demand serves to pull innovation is used. In contrast, the push-funding model is mainly used for products where the market is deficient or for early-stage research with uncertain or unpatentable results. *When given the choice between pull and push*, *pull mechanisms dominate thoroughly*.

### Demand enhancement mechanisms

Broadly speaking, the difference between the approach to funding drugs for neglected diseases and drugs for global diseases is that the former receive grants to the point of completion; grant-type funding for the latter is present mainly in the early stages of research, and the funding gap is made up through commercial investors who seek a return through sales. It is clear that the commercial investor concept cannot be applied for neglected diseases – or generally for poverty-related diseases – because effective demand is too low. Using grant-type funding from beginning to end appears to be the solution only when effective demand is insufficient.

There is, however, an alternative approach to push funding: demand enhancement mechanisms. When market demand fails to create sufficient incentives for private investment, *demand enhancement* can fill the gap. The classic example of this in neglected diseases is the Pneumococcal Advanced Market Commitment (AMC), which offered supplementary payments for the delivery of pneumococcal vaccines to patients in low-income countries. The model was simple: for each qualifying vaccine delivered below a given price point ($3.50), manufacturers would be paid a supplement ($3.50). Market demand was insufficient to support the expansion of manufacturing; enhancement by the AMC created more confidence that the manufacturers could earn profits by supplying this market. Although the AMC has been effective in achieving its goals in vaccination against pneumonia, the model has yet to be replicated more broadly [11]. There are, however, arguments that the approach would be even more productive if applied to more “technologically distant” targets, i.e., to support drug development from an earlier stage [29].

High-income country governments appear to have decided that demand enhancement is the right approach for procuring drug innovation for products to treat their own populations. Indeed, we precisely see this in the antibiotics market, where governments are actively establishing prize-type mechanisms because the effective demand is too low to stimulate investment into antibiotic research and development. The reasons for the failure of demand are complex but very apparent in outcomes: the amount of investment in antibiotics has plummeted while numerous small firms that brought new antibiotics to market have fallen into bankruptcy [30]. If antibiotics were treated similarly to drugs for neglected diseases, the response would be straightforward: governments could simply provide grants to a PDP to bring new products to market. Indeed, this path – nationalization of antibiotics research – has been proposed by Singer, Kirchhelle, and Roberts [31], and has apparently been rejected.^1^ Instead, there has been an attempt to enhance the market so that commercial firms are rewarded for developing new antibiotics. The rewards are, of course, related to the estimated value, just as in an ordinary market [34]. The UK has moved the furthest in this direction, with its antibiotic subscription pilot now followed up with a proposal for expanding, extending, and improving the approach. A similar approach has been proposed by Japan [35]. In the US, the Pasteur Act has been advanced to provide similar market entry payments, and in the EU, there is a multi-year process that involves “the design and governance of a Union multi-country pull incentive scheme” [36]. In effect, where market demand has been found insufficient, high-income country governments have combined investments in “push” for pre-clinical research with “pull” schemes to enhance demand, allowing for competitive commercial clinical development programs.

Demand enhancement was also used, along with push funding, to support the rapid development of vaccines and therapeutics during the COVID-19 pandemic. There were certainly substantial subsidies granted to some firms during the vaccine development process, and the success of these efforts has been characterized as evidence that there should be much deeper government involvement in the pharmaceutical industry [37]. However, most funding was performance-based / pull, in the sense that it was conditional on the approval and delivery of vaccines [38]. Indeed, the four largest manufacturers are said to have earned profits of $90bn from their covid vaccines, a sum that dwarfs any push funding [39]. 

Insurance is perhaps the most widespread form of demand enhancement. It used to be that rare diseases were a neglected market because small patient populations made them unattractive. But with extremely high prices made possible by the risk-pooling property of insurance, the effective demand for treatments for rare diseases is now sufficient to incentivize investment into these diseases, and there has been a substantial growth in the number of effective (and high-priced) orphan and ultra-orphan drugs. (As Horgan et al. (2020) note, however, 95% of rare diseases still lack a treatment option [40]).

Pull funding is the preferred model in rich countries for bringing drugs through clinical trials and to market, even when effective demand is low, and using pull funding requires the creation of a market-enhancement mechanism. So, one has to ask why, when it comes to advancing new drugs for neglected diseases for populations in the global south, the choice is to use push funding all the way through to regulatory approval. One possible reason for using push funding is that these poorly developed markets lack effective demand, as discussed above. However, there are numerous established models that could be used to enhance market demand for neglected diseases, such as advanced market commitments and subscription models, as well as innovative mechanisms such as Senator Sanders Medical Innovation Prize Fund [41] and the Health Impact Fund [42]. These latter two mechanisms propose to “delink” payment for innovative medicines from the price paid by the consumer, so that companies would earn profits based on achieved health benefits, while prices would be based on generic costs of manufacture. This approach could provide a pull incentive for development of neglected diseases.

## Conclusion

As discussed in Sect. 3 above, it seems clear that both push and pull mechanisms have advantages and disadvantages given specific circumstances. What is surprising is that, when it comes to clinical development of new drugs, the choice between push or pull funding appears to be based on the patient's income. As the approach to antibiotics shows, governments in high-income countries prefer to use the “pull” approach. They are willing to create an artificial market designed to induce private investment when ordinary commercial demand is inadequate. However, these same governments almost exclusively support the “push” approach for drugs intended to treat mainly poor people. What is the reason for these different approaches? If push funding is more efficient, it should be used widely, including to bring new drugs for global diseases to market. Drugs for Neglected Disease Initiative (DNDi), a not-for-profit PDP, claims it can develop new drugs for about one-tenth the cost of industry [43]. If the same efficiency could be achieved for global diseases, we could have much more innovation with lower prices for everyone. Some observers have even argued for a much larger role of government in drug research, including nationalization of the industry [44]. On the other hand, if pull funding is more efficient, it seems strange that push funding is used to address the needs of the most vulnerable people. A critical distinction between push funding and demand enhancement is that with most push funding, as noted above, it is possible to direct the support to favored research institutions.

The inconsistent use of push and pull funding suggests that there would be enormous value in experimenting more aggressively. This would involve more push funding for clinical trials of global diseases and more pull funding for neglected diseases. Push funding for global diseases could include more government funding of clinical trials. Pull funding for neglected diseases would include using demand enhancement mechanisms, such as advanced market commitments or the Health Impact Fund, to support commercial investment into the development of drugs for which market demand is disproportionately low compared to the potential health benefits. Given the billions of dollars spent annually on pharmaceutical research, and the immense potential for new drugs to improve human well-being, there is an urgent need to know what works best.

## Data Availability

N/A.

## Abbreviations

AMC: Advance Market Commitment
PDP: Product Development Partnership

## Footnotes

1 Not everyone agrees that governments should use pull incentives to support the development of new antibiotics, of course [32, 33].
